# Knowledge, Attitudes, and Compliance About Infection Prevention and Control in Nursing Students: A Systematic Review

**DOI:** 10.3390/diseases14070263

**Published:** 2026-07-21

**Authors:** Ana De Maya-Martínez, Omar Cauli, María del Carmen Giménez-Espert, Cristina Buigues

**Affiliations:** Department of Nursing, University of Valencia, 46010 Valencia, Spain; ana.maya@uv.es (A.D.M.-M.); maria.c.gimenez@uv.es (M.d.C.G.-E.); cristina.buigues@uv.es (C.B.)

**Keywords:** hand hygiene, infection prevention and control, healthcare-associated infections, knowledge, nursing students, standard precautions

## Abstract

Background: Healthcare-associated infections (HAIs) remain a major preventable challenge in healthcare systems worldwide. Nursing students play a key role in infection prevention during clinical training; therefore, adequate education in hand hygiene (HH), standard precautions (SPs), and infection prevention and control (IPC) is essential. This systematic review aimed to evaluate nursing students’ knowledge of HAI prevention according to international recommendations and to identify factors associated with higher knowledge levels. Methods: A systematic review was conducted following Joanna Briggs Institute methodology and PRISMA guidelines. Searches were performed in MEDLINE (PubMed), CINAHL, Cochrane Library, Embase, Scopus, and Web of Science. Observational studies assessing nursing students’ knowledge of HH, SPs, or IPC were included. Methodological quality was assessed using the Joanna Briggs Institute critical appraisal checklist for analytical cross-sectional studies. Results: Fifty-two studies involving 13,912 nursing students across five continents were included. Most studies focused on HH, followed by SPs and IPC. The WHO Five Moments for Hand Hygiene (14 of 26 (53.8%)) was the most frequently used instrument for HH assessment, while the Compliance with Standard Precautions Scale (5/11 (45.5%)) was the most used for SPs. Regarding IPC, most studies (5/7 (71.4%)) evaluated it by study-specific questionnaires developed/adapted by the researchers. Higher knowledge and compliance levels were generally associated with advanced academic year, prior training, positive attitudes, and supportive clinical environments. However, substantial heterogeneity was identified across study designs, assessment instruments, outcome measures, and methodological quality. Conclusions: Continued and updated education in HH, SPs, and IPC appears essential for improving nursing students’ knowledge and preventive practices. Greater standardization of assessment methods is needed to improve comparability across studies.

## 1. Introduction

Healthcare-associated infections (HAIs) are infections acquired while receiving healthcare for another condition in any healthcare setting, including hospitals, outpatient facilities, and home care environments [1,2]. HAIs remain one of the most important preventable patient safety challenges worldwide. In Europe alone, HAIs affect between 4.5 and 5 million patients annually and are associated with prolonged hospitalisation, increased healthcare costs, and the growing global threat of antimicrobial resistance [3]. Both the World Health Organization (WHO) and the European Centre for Disease Prevention and Control (ECDC) estimate that a substantial proportion of HAIs are preventable through appropriate infection prevention measures [1,3]. Consequently, strengthening infection prevention protocols and educational strategies remains a major international healthcare priority [1,3,4].

Infection prevention and control (IPC) constitute a broad organisational, educational, and safety-oriented framework aimed at reducing the risk of healthcare-associated infections (HAIs). Within this framework, standard precautions (SPs) represent the basic set of preventive measures that should be applied to all patients, regardless of infection status, and include hand hygiene (HH), appropriate use of personal protective equipment (PPE), safe handling of sharps, environmental decontamination, respiratory hygiene, waste management, and prevention of cross-transmission [1,5,6]. Among these measures, HH is one of the most widely studied and evidence-based components, given its central role in reducing microorganism transmission in healthcare settings. The WHO defines HH as any action aimed at cleaning the hands to reduce microbial contamination [1,2], and its central role in interrupting microorganism transmission explains why it has been extensively addressed in international recommendations and empirical studies. In this review, HH, SPs, and broader IPC were considered analytically distinct but conceptually interrelated subdomains, reflecting how the available literature and assessment instruments are commonly organised: HH as a key component of SPs, SPs as the basic clinical framework for infection prevention, and IPC as the wider organisational and educational context in which these measures are implemented [7,8,9,10].

Nurses play a central role in the prevention and control of HAIs because of their continuous and direct contact with patients during clinical care [11,12]. Consequently, nursing students (NSs) must acquire adequate knowledge and competencies in HH, SPs, and IPC during their academic and clinical training. Improving knowledge in these areas may contribute to reducing microorganism transmission, minimising healthcare costs and workload, and improving the quality and safety of patient care [13,14,15,16,17,18]. However, most previous studies involving NSs have focused primarily on HH, with comparatively fewer studies addressing SPs or broader IPC concepts [19,20,21,22,23,24,25,26,27,28,29]. In addition, much of the available evidence relies on self-reported questionnaires rather than direct observation of clinical compliance.

To date, few reviews have simultaneously examined HH, SPs, and IPC knowledge among NSs from a comprehensive perspective aligned with international recommendations. While a recent review [30] focused primarily on nursing students’ knowledge of healthcare-associated infections and infection prevention, the present review offers a broader and more comprehensive perspective on infection prevention and control (IPC) competence. Specifically, it examines IPC knowledge as the primary outcome while also synthesising related constructs frequently reported in the literature, including attitudes, perceptions, self-reported practices, adherence, and compliance. In addition, we systematically analysed the assessment instruments used to evaluate these domains and identified the factors associated with higher knowledge levels.

Therefore, the primary objective of this systematic review was to analyse nursing students’ knowledge of infection prevention and control in relation to HH, SPs, and broader IPC recommendations. The secondary objectives were to identify the instruments most commonly used to assess these domains, to examine the factors associated with higher knowledge levels, and to narratively synthesise related outcomes, including attitudes, perceptions, self-reported practices, adherence, and compliance, when reported by the included studies. By synthesising the available evidence, this review may support healthcare educators, academic institutions, and policymakers in the development of more effective infection prevention training strategies and educational policies [31].

## 2. Materials and Methods

**Figure 1:**
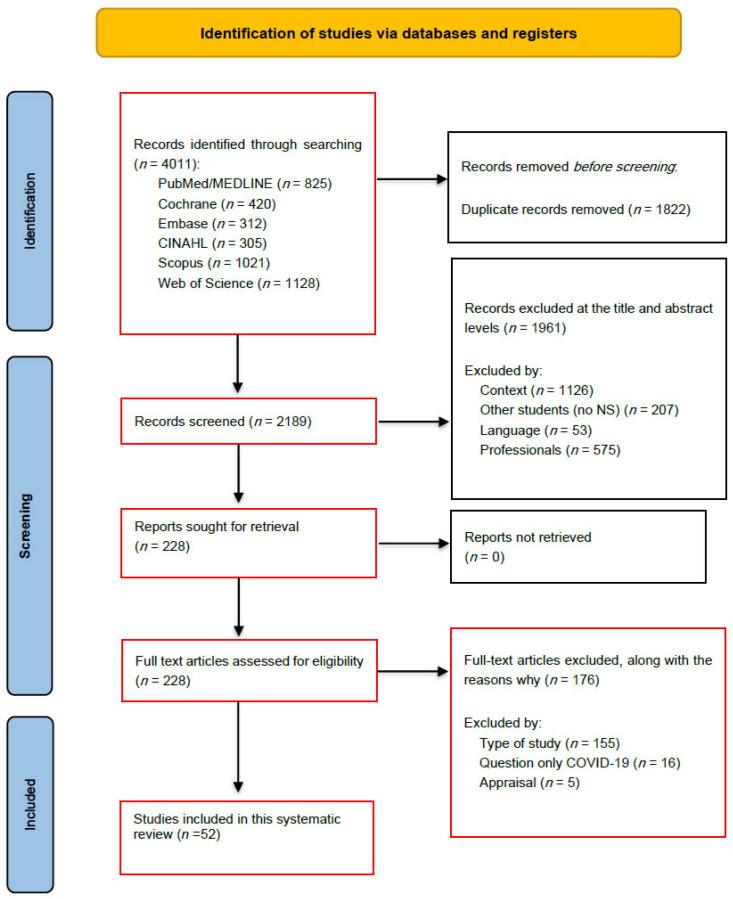
Flow chart. Abbreviations: NS, nursing student; COVID-19, coronavirus disease 2019.

This systematic review included studies assessing nursing students’ (NSs) knowledge of hand hygiene (HH), standard precautions (SPs), and infection prevention and control (IPC), as well as the instruments used to measure these domains and the variables associated with knowledge levels. The review was conducted in accordance with the Joanna Briggs Institute (JBI) methodology for systematic reviews of evidence synthesis [32,33]. Reporting of the review process followed the Preferred Reporting Items for Systematic Reviews and Meta-Analyses (PRISMA) 2020 guidelines, and the study selection process is presented in a PRISMA flow diagram (Figure 1) [34]. The review protocol was not registered in PROSPERO.

### 2.1. Search Strategy

The research question was structured according to the PEO framework (Population–Exposure–Outcome), which is appropriate for observational evidence synthesis. The Population was undergraduate nursing students; the Exposure comprised infection prevention-related domains, including hand hygiene (HH), standard precautions (SPs), infection prevention and control (IPC), personal protective equipment (PPE), and related educational or clinical exposure; and the primary Outcome was knowledge of infection prevention. Attitudes, perceptions, self-reported practices, adherence, and compliance were considered related secondary outcomes when reported by the included studies.

The search strategy was designed to identify studies assessing nursing students’ knowledge of HH, SPs, IPC, PPE, and related infection prevention measures. The following core search syntax was applied and adapted to each database: (((nurs* student* OR nursing student*) AND (hand hygiene OR standard precaution* OR infection prevention and control OR infection prevention OR nosocomial infection* OR healthcare-associated infection* OR antisepsis OR personal protective equipment OR PPE OR glove* OR mask* OR gown* OR goggle*) AND (knowledge))).

The literature search was conducted in MEDLINE via PubMed, CINAHL via EBSCOhost, Cochrane Library, Embase, Scopus, and Web of Science. Controlled vocabulary terms, including MeSH and Emtree terms where applicable, were combined with free-text keywords using Boolean operators. The final search was performed on 7 January 2026. Screening, full-text assessment, and selection of eligible studies were subsequently conducted between January and April 2026. The complete database-specific search strategies, including controlled vocabulary terms, free-text terms, field tags, Boolean operators, filters, limits, language restrictions, final search date, and the number of records retrieved from each database, are provided in Appendix A.

Only peer-reviewed full-text articles published in English or Spanish were eligible for inclusion. Grey literature was not searched. Although Spanish-language publications were eligible, the search syntax was applied in English; therefore, potentially relevant studies indexed only with Spanish-language terms may not have been retrieved.

The search yielded 4011 records. After duplicate removal (*n* = 1822), studies were screened according to the predefined inclusion and exclusion criteria. The study selection process is presented in the PRISMA 2020 flow diagram (Figure 1) [34].

### 2.2. Inclusion and Exclusion Criteria

To address the objectives of this review, studies were eligible for inclusion if they were original, peer-reviewed, full-text articles published in English or Spanish that assessed nursing students’ (NSs) knowledge, attitudes, or compliance related to hand hygiene (HH), standard precautions (SPs), or infection prevention and control (IPC). Cross-sectional observational studies using questionnaires, validated scales, or structured survey instruments were included.

Studies focusing exclusively on registered nurses, healthcare professionals, or students from disciplines other than nursing were excluded. Reviews, editorials, conference abstracts, protocols, and studies without sufficient methodological information were also excluded.

Studies focusing exclusively on infection prevention knowledge or practices related to SARS-CoV-2 were excluded because the objective of this review was to evaluate general infection prevention competencies among undergraduate nursing students, rather than competencies specific to a single infectious disease.

### 2.3. Study Selection

The Zotero reference management system (version 6.0.37) was used to organise citations, manage duplicate records, and support the screening process. Potentially relevant studies were retrieved in full text, and citation details were imported into the Joanna Briggs Institute System for the Unified Management, Assessment and Review of Information (JBI SUMARI) for data extraction and methodological appraisal [32].

Data extracted from the included studies comprised study setting, design, participant characteristics (including mean age, sex distribution, and academic year), assessment instruments, and variables associated with knowledge of hand hygiene (HH), standard precautions (SPs), or infection prevention and control (IPC) (Appendix B). The standardised JBI SUMARI extraction template was used throughout the review process. The study selection procedure and reasons for exclusion were documented according to PRISMA 2020 recommendations and are presented in the PRISMA flow diagram (Figure 1) [34].

### 2.4. Assessment of the Methodological Quality

Methodological quality was independently assessed by two reviewers (A.d.M.M. and C.B.) using the Joanna Briggs Institute (JBI) Critical Appraisal Checklist for Analytical Cross-Sectional Studies. Disagreements during the appraisal process were resolved through discussion with a third reviewer (M.d.C.G.-E.).

The checklist consists of eight items evaluating methodological rigour, including sampling procedures, measurement validity, confounding factors, and statistical analysis. Each item was scored as 1 (“yes”) or 0 (“no”, “unclear”, or “not applicable”). For the purposes of this review, studies scoring > 70% were classified as high quality, scores between 50% and 70% as moderate quality, and scores < 50% as low quality. Only studies classified as high or moderate quality were included in the review. The methodological appraisal results are presented in Appendix C.

To improve methodological consistency, study screening and quality appraisal were conducted independently by the reviewers, and discrepancies were resolved by consensus.

## 3. Results

Following the study selection process described in the PRISMA 2020 flow diagram (Figure 1), 52 studies were included in the review [31]. The studies represented a broad geographical distribution across Asia (*n* = 29), Europe (*n* = 12), Africa (*n* = 6), Oceania (*n* = 3), and the Americas (*n* = 2). Overall, the included studies involved 13,912 nursing students (NSs), most of whom were female (75.20%), with mean ages ranging between 20 and 25 years. Sample sizes varied considerably across studies, ranging from 14 to 829 NS participants. Most studies included students from multiple academic years to improve sample representativeness.

### 3.1. Characteristics of the Included Studies

The included studies investigated infection prevention from different perspectives, encompassing hand hygiene, standard precautions, infection prevention and control, healthcare-associated infections, personal protective equipment, and occupational exposure. Although all studies addressed infection prevention among nursing students, they evaluated different outcome constructs using a wide variety of assessment instruments.

Given this heterogeneity in both study objectives and outcome measures, the findings were synthesised according to the principal construct evaluated rather than according to the infection prevention topic addressed.

Knowledge was the most frequently investigated construct, followed by attitudes, perceptions, self-reported practices, adherence, and compliance. Several studies evaluated more than one construct simultaneously, whereas others focused exclusively on a single outcome. Accordingly, the findings are presented according to the principal construct assessed in each study: knowledge, attitudes, perceptions, self-reported practices, adherence, compliance, and assessment instruments.

### 3.2. Knowledge

Knowledge was the most frequently assessed construct across the included studies and was evaluated in relation to hand hygiene (HH), standard precautions (SPs), infection prevention and control (IPC), or broader infection prevention topics. Overall, 33 studies assessed infection prevention knowledge using a wide range of validated and ad hoc instruments. As shown in Table 1, substantial heterogeneity was observed in the assessment instruments, scoring systems, and criteria used to classify and interpret knowledge levels across the included studies. This methodological variability limited direct comparison of findings between studies. Only a small number of studies applied predefined cut-off values to classify knowledge as poor, moderate or good, whilst the majority relied on qualitative interpretations defined by the authors, descriptive statistics or comparisons between groups of participants without explicit classification thresholds. Despite these methodological differences, the quantitative results generally indicated moderate, albeit variable, levels of knowledge amongst nursing students. This overall pattern was also reflected in studies reporting generally sufficient knowledge of standard precautions and infection prevention, despite persistent deficiencies in specific domains such as sharps management and healthcare workers’ care. Likewise, lower infection prevention knowledge scores observed among nursing students compared with medical students further highlight the need to strengthen infection prevention education early in undergraduate curricula [35,36].

Quantitative knowledge outcomes varied considerably across infection prevention domains and assessment instruments. In hand hygiene studies, reported knowledge ranged from 68.1% correct responses on a 21-item questionnaire [15] to a mean score of 4.59 ± 0.72 out of 6 on the WHO Hand Hygiene Questionnaire, with 80% of students scoring above four points [23]. Among studies applying WHO-based cut-off criteria, Thakker and Jadhav reported that only 7.5% of participants achieved good hand hygiene knowledge, whereas 69.1% were classified as having moderate knowledge [37]. Similarly, Nair et al. found that only 9% of participants achieved good hand hygiene knowledge [38]. In studies assessing standard precautions, reported knowledge scores included a mean score of 13.8 ± 3.3 out of 18 [39] and a mean percentage score of 65.0% [40], while Ayele et al. reported that only 32.4% of students demonstrated good knowledge of standard precautions [41]. Broader IPC studies also showed variable results, including a mean knowledge score of 12.49 ± 2.3 out of 18 for nosocomial infection control standards [42] and a mean score of 51.53 ± 0.89 out of 100 on the Infection Control Standardized Questionnaire, with only 15.5% of students achieving a score of ≥70/100 [19]. Comparable findings were also reported, with moderate knowledge scores positively correlated with infection prevention performance [43].

Although female sex was associated with higher knowledge scores in some studies, this finding should be interpreted cautiously, as the included samples were predominantly female, reflecting the sex distribution of undergraduate nursing programmes. Despite these associations, recurrent knowledge deficits were identified across infection prevention domains, particularly in relation to recognising the patient as a primary source of HAI-related pathogens, understanding the role of healthcare workers’ hands in microorganism transmission, identifying the appropriate indications and duration of alcohol-based hand rub use, applying the WHO Five Moments for Hand Hygiene, understanding transmission-based precautions, managing occupational exposure incidents, and selecting appropriate personal protective equipment. Similar deficiencies were also identified, with persistent gaps in students’ understanding of hand hygiene and standard precaution principles despite previous theoretical instruction [44,45]. Conversely, nursing students generally demonstrated better knowledge of glove use, hand hygiene after exposure to body fluids, and the fundamental principles of standard precautions.

**Table 1:** Approaches used to interpret knowledge levels across the included studies.

Interpretation Approach	Description	Representative Studies
Authors’ qualitative interpretation	Knowledge classified qualitatively (e.g., good, moderate, satisfactory, adequate) without explicit cut-off values	[15,25,41,46,47,48]
Explicit cut-off values	Knowledge classified using predefined percentage thresholds	[37,38]
Comparative analysis	Knowledge compared between academic years, sexes or disciplines without overall classification	[19,28,49,50]
Descriptive analysis	Item-level knowledge reported without global categorisation	[7,40]
Correlational/predictive analysis	Knowledge analysed as an explanatory variable associated with performance or behavioural outcomes	[42,51,52]

### 3.3. Attitudes, Perceptions and Self-Reported Practices

Compared with knowledge, attitudes, perceptions, and self-reported practices were evaluated in fewer studies and were commonly assessed using multidimensional questionnaires incorporating several infection prevention constructs simultaneously. These outcomes were investigated in relation to hand hygiene (HH), standard precautions (SPs), and broader infection prevention and control (IPC) topics, with most studies assessing more than one construct within the same instrument rather than evaluating them independently.

Overall, nursing students demonstrated positive attitudes towards infection prevention and recognised the importance of hand hygiene, standard precautions, and the appropriate use of personal protective equipment. More favourable attitudes were consistently associated with higher knowledge levels, previous infection prevention education or training, and greater confidence in infection prevention practices. Likewise, positive perceptions of the clinical learning environment were associated with safer workplace environments, institutional support, and previous IPC training. More positive perceptions of hand hygiene were associated with better self-reported hand hygiene performance, reinforcing the relationship between knowledge, perceptions, and preventive behaviours [43]. Conversely, observing poor infection prevention practices among healthcare professionals, perceived organisational barriers, and unsafe clinical environments negatively influenced students’ perceptions and their intention to comply with recommended infection prevention measures [14,15,24,25,41,46,52,53,54,55,56]. In addition, nursing students generally expressed positive attitudes towards patient participation in hand hygiene promotion, with many considering reminders from patients and relatives about hand hygiene to be acceptable and supportive of safer care [57].

Self-reported infection prevention practices were generally favourable, although considerable variability was observed across specific behaviours. Nursing students consistently reported performing hand hygiene more frequently after exposure to body fluids, following patient contact, and after toilet use than before patient contact, before aseptic procedures, or before glove use. These findings are consistent with evidence showing high overall self-reported hand hygiene practice scores, although lower compliance was observed before glove use than after patient contact [58]. Frequently reported barriers to appropriate infection prevention practices included forgetting to perform hand hygiene, time pressure during clinical placements, inadequate availability of resources, and the influence of healthcare professionals’ behaviour as clinical role models [7,26,27,58,59,60,61].

### 3.4. Adherence and Compliance

Compliance with infection prevention measures, and less frequently adherence, were evaluated in a smaller number of studies, primarily in relation to standard precautions and hand hygiene during clinical placements. Most studies assessed compliance using validated instruments, particularly the Compliance with Standard Precautions Scale (CSPS), whereas adherence was mainly explored through questionnaires examining factors influencing compliance with standard precautions. One study also assessed hand hygiene practices using the validated Hand Hygiene Practices Inventory (HHPI), providing a more behaviour-focused evaluation of students’ routine hand hygiene practices [58].

Overall, nursing students demonstrated moderate to high levels of compliance, although performance varied according to the specific infection prevention measure assessed. Higher compliance was consistently reported for safe sharps disposal, hand hygiene following exposure to body fluids, appropriate waste management, and the use of gloves and face masks when indicated. In contrast, lower compliance was observed for hand hygiene before patient contact, before aseptic procedures, the use of protective gowns and eye protection, and environmental decontamination practices [7,20,22,41,59]. Similarly, high overall self-reported hand hygiene practice scores were reported, although lower compliance was observed before glove use than after patient contact, supporting the pattern identified across the included studies [58].

Higher compliance was associated with more advanced academic year, previous infection prevention education or training, greater knowledge of infection prevention measures, and supportive clinical learning environments. Conversely, limited availability of resources, inadequate supervision during clinical placements, perceived organisational barriers, and poor role modelling by healthcare professionals were the most frequently reported factors associated with lower compliance [7,52,55,60].

### 3.5. Assessment Instruments

A wide variety of instruments was used to assess infection prevention competencies among nursing students, reflecting considerable heterogeneity in both the constructs evaluated and the measurement approaches employed (Table 2). Validated questionnaires were predominantly used to assess hand hygiene and standard precautions, whereas studies investigating broader infection prevention and control (IPC) competencies more frequently relied on ad hoc questionnaires developed for individual studies.

Among studies evaluating hand hygiene, the most frequently used instruments were questionnaires based on the World Health Organization (WHO) Five Moments for Hand Hygiene framework, followed by the Hand Hygiene Questionnaire (HHQ), the Hand Hygiene Practices Inventory (HHPI), and the Hand Hygiene Beliefs Scale (HHBS). Together, these instruments assessed a range of constructs, including knowledge, beliefs, attitudes, and self-reported practices [23,56,61,62]. In addition, a modified WHO Hand Hygiene Questionnaire was used to assess knowledge, perceptions, and performance simultaneously, whereas the validated Hand Hygiene Practices Inventory (HHPI) was used to assess self-reported hand hygiene practices among nursing students [43,58].

Studies focusing on standard precautions primarily used validated instruments such as the Compliance with Standard Precautions Scale (CSPS), the Factors Influencing Adherence to Standard Precautions Scale–Student Version (FIASPS-SV), the Questionnaire on Knowledge of Standard Precautions (QKSP), and the Questionnaire for Compliance with Standard Precautions (QCSP), allowing separate assessment of compliance, determinants of adherence, and knowledge of standard precautions [22,41,59,63]. Other studies used adapted questionnaires based on previously published instruments. For example, an adapted 41-item questionnaire assessing five domains of standard precautions and infection prevention knowledge and a questionnaire adapted from the Rouen University infection prevention survey were used to evaluate students’ knowledge of standard precautions [35,36].

In contrast, relatively few validated instruments were available for broader IPC competencies. The Infection Control Standardized Questionnaire (ICSQ) and the Leading a Culture of Quality for Infection Prevention (LCQ-IP) were the only validated IPC-specific instruments identified. Most studies investigating broader IPC competencies relied on ad hoc questionnaires that simultaneously assessed multiple constructs, including knowledge, attitudes, perceptions, self-reported practices, adherence, and compliance [7,19,55]. Similarly, infection prevention knowledge was assessed using investigator-developed questionnaires focusing on hand hygiene and standard precautions, reflecting the continued reliance on study-specific instruments within this field [44,45].

Overall, the variability in assessment instruments, together with the frequent use of study-specific questionnaires assessing multiple constructs simultaneously, highlights the lack of a standardised approach for evaluating infection prevention competencies among nursing students.

## 4. Discussion

### 4.1. Overview

The present review extends the existing evidence on infection prevention and control (IPC) education among nursing students [30] by providing a broader and more comprehensive synthesis than previous reviews by analysing related constructs such as students’ attitudes, perceptions, self-reported practices, adherence, and compliance. We also systematically examined the assessment instruments used to evaluate these domains and identified the factors associated with higher knowledge levels. By integrating these complementary dimensions, our review contextualises IPC knowledge within the broader educational and behavioural factors that may influence infection prevention competence among nursing students.

Overall, the findings indicate that nursing students generally demonstrate moderate to satisfactory knowledge, positive attitudes towards infection prevention, and moderate to high levels of self-reported compliance with recommended IPC measures. However, important knowledge gaps persist in several core IPC concepts, and compliance remains inconsistent across specific preventive behaviours. Furthermore, this review identified substantial methodological heterogeneity not only in the assessment instruments used to evaluate infection prevention competencies but also in the approaches adopted to classify and interpret knowledge levels. Together, these methodological differences limit direct comparisons between studies and highlight the need for greater standardisation of both educational assessment and reporting methods.

### 4.2. Knowledge

Overall, the available evidence suggests that infection prevention and control (IPC) knowledge appears to develop progressively throughout undergraduate nursing education, although important gaps remain in several core IPC concepts. Higher knowledge levels were consistently reported among senior nursing students and those who had received previous IPC education or recent clinical training, suggesting that repeated exposure to theoretical instruction and supervised clinical practice contributes to the progressive development of IPC competencies [15,23,49,61,64]. This interpretation is further supported by evidence highlighting the need to strengthen infection prevention education early in undergraduate nursing curricula to ensure the progressive acquisition of IPC competencies [36].

Despite these improvements, knowledge deficiencies remained remarkably consistent across the included studies and were mainly related to fundamental IPC concepts, including microorganism transmission, the WHO Five Moments for Hand Hygiene, the appropriate use of alcohol-based hand rubs, transmission-based precautions, post-exposure management, and the correct use of personal protective equipment. These persistent gaps suggest that, although knowledge increases with academic progression, important IPC concepts are not consistently consolidated during undergraduate education [15,37,47,56,64]. Comparable deficiencies were also reported, indicating that theoretical instruction alone may be insufficient to ensure the long-term retention and application of core infection prevention principles [44,45].

An important methodological finding of the present review, supported by the comparative analysis presented in Table 1, was the considerable heterogeneity in the approaches used to assess and interpret IPC knowledge across studies. While only a limited number of studies classified knowledge using predefined cut-off values, most relied on author-defined qualitative interpretations or descriptive comparisons without explicit classification criteria. Consequently, direct comparison of reported knowledge levels should be interpreted cautiously. This methodological heterogeneity highlights the need for greater standardisation of both assessment instruments and approaches used to classify IPC knowledge among nursing students.

The findings of this review are consistent with the recent systematic review by Giordano et al. [30], which also reported progressive improvements in nursing students’ IPC knowledge alongside persistent educational gaps across key infection prevention domains. Together, these findings support the view that IPC education should be considered a longitudinal educational process rather than a series of isolated learning activities. Continuous educational reinforcement throughout undergraduate nursing programmes, aligned with the World Health Organization Core Components of Infection Prevention and Control Programmes [65], may facilitate the consolidation of theoretical knowledge and its translation into safe clinical practice. This interpretation is also supported by evidence demonstrating positive associations between infection prevention knowledge, perceptions, and self-reported performance, suggesting that educational interventions should simultaneously address cognitive, behavioural, and contextual dimensions [43]. Future research would also benefit from the adoption of more standardised assessment instruments and explicit knowledge classification criteria, facilitating comparisons across educational settings and strengthening the evidence base for undergraduate IPC education.

### 4.3. Attitudes, Perceptions and Self-Reported Practices

Although nursing students generally demonstrated positive attitudes towards infection prevention and recognised the importance of infection prevention and control (IPC) measures, the findings of this review suggest that favourable attitudes alone are insufficient to ensure the consistent adoption of safe infection prevention behaviours. Positive attitudes were frequently associated with higher levels of IPC knowledge and previous infection prevention education or training, supporting the view that knowledge provides an important foundation for the development of infection prevention competencies [15,25,46,54,56]. However, several studies demonstrated that adequate knowledge did not consistently translate into appropriate self-reported practices, indicating that infection prevention behaviours are influenced by factors extending beyond knowledge acquisition alone. This interpretation is further supported by evidence showing that, although nursing students generally demonstrated good hand hygiene knowledge, attitudes and self-reported practices remained comparatively less favourable, reinforcing the need for educational approaches that target behavioural as well as cognitive aspects of infection prevention [66].

One of the most consistent findings of this review was the influence of the clinical learning environment on nursing students’ infection prevention behaviours. Positive perceptions of institutional support, effective supervision, and a strong patient safety culture were associated with more favourable self-reported practices, whereas observing poor infection prevention behaviours among healthcare professionals, inadequate supervision, organisational barriers, and limited availability of resources negatively influenced students’ perceptions and reduced their willingness to adopt recommended IPC practices [24,49,52,54,55]. Similarly, the importance of a supportive safety culture was reinforced by findings showing that nursing students generally welcomed patient participation in hand hygiene promotion, suggesting that open communication and shared responsibility may further strengthen infection prevention behaviours during clinical training [57]. These findings reinforce the importance of positive clinical role models and suggest that infection prevention competencies are shaped not only through formal education but also through the clinical learning environment, where students observe, practise, and reinforce recommended IPC behaviours during clinical placements.

Taken together, these findings indicate that the development of infection prevention competencies extends beyond the acquisition of theoretical knowledge. Undergraduate nursing curricula should therefore adopt a longitudinal competency-based approach that integrates knowledge acquisition, positive attitudes, behavioural skills, reflective practice, and supportive clinical learning environments throughout the educational process. This interpretation is consistent with the World Health Organization Core Components of Infection Prevention and Control Programmes [65], which recognise education, training, and institutional safety culture as complementary elements required to promote sustainable infection prevention behaviours. Similar conclusions have also been reported in a recent systematic review [30], supporting the importance of combining theoretical instruction with repeated clinical reinforcement to facilitate the translation of knowledge into safe clinical practice. Furthermore, evidence suggests that even when nursing students demonstrate favourable self-reported hand hygiene practices, adherence may still vary according to specific clinical situations, reinforcing the need for continuous behavioural reinforcement throughout undergraduate education [58].

### 4.4. Adherence and Compliance

Although nursing students generally demonstrated moderate to high levels of compliance with recommended infection prevention and control (IPC) measures, compliance varied according to the specific preventive behaviour evaluated. Higher compliance was consistently reported for practices related to safe sharps disposal, hand hygiene following exposure to body fluids, and the use of gloves or face masks when indicated, whereas lower compliance was observed before patient contact, before aseptic procedures, and for the use of gowns and eye protection [7,20,22,41,59]. Comparable findings have also been reported, with consistently higher self-reported hand hygiene practices after patient contact than before glove use, suggesting that behaviours associated with more immediate or visible risks are more readily adopted by nursing students [58]. This pattern suggests that preventive behaviours associated with immediate and clearly perceived risks may be adopted more consistently than those primarily intended to prevent cross-transmission between patients.

An important methodological finding of this review was that, unlike knowledge, adherence and compliance were assessed using a relatively small number of well-established and validated instruments. Most studies evaluating standard precautions employed the Compliance with Standard Precautions Scale (CSPS), whereas studies exploring behavioural determinants primarily used the Factors Influencing Adherence to Standard Precautions Scale–Student Version (FIASPS-SV) [22,29,59]. The additional use of the validated Hand Hygiene Practices Inventory (HHPI) further supports the availability of robust instruments for assessing self-reported infection prevention behaviours among nursing students [58]. This greater methodological consistency facilitated comparisons across studies and contrasts with the substantial heterogeneity identified in the assessment and interpretation of IPC knowledge.

Taken together, these findings suggest that improving compliance requires more than increasing theoretical knowledge alone. Consistent implementation of infection prevention measures appears to depend on the interaction between knowledge, repeated supervised clinical practice, constructive feedback, positive clinical role modelling, and supportive learning environments [7,52,55,60]. Future research would benefit from maintaining the use of validated compliance instruments while further investigating the behavioural and organisational factors influencing nursing students’ adherence to IPC measures across different educational and clinical settings.

### 4.5. Assessment Instruments

An important contribution of this review was the comprehensive analysis of the instruments used to evaluate infection prevention and control (IPC) competencies among nursing students. Although several validated questionnaires were identified, particularly for hand hygiene and standard precautions, the assessment of broader IPC competencies relied predominantly on study-specific instruments developed for individual investigations [7,19,22,23]. In addition, several studies employed questionnaires adapted from previously published instruments rather than fully validated IPC-specific tools, including modified WHO-based questionnaires and other investigator-adapted instruments [35,36,43,49]. This variability reflects the absence of a universally accepted framework for evaluating IPC competencies in undergraduate nursing education and makes direct comparison of findings across studies challenging.

Beyond differences in the instruments themselves, substantial variability was also observed in the constructs assessed. While some questionnaires focused exclusively on knowledge or compliance, others simultaneously evaluated multiple dimensions, including knowledge, attitudes, perceptions, self-reported practices, adherence, and compliance [46,49,52,55]. The use of investigator-developed, adapted, and modified instruments across studies further illustrates the lack of consensus regarding which IPC competencies should be prioritised and how they should be measured. This heterogeneity suggests not only a lack of standardisation in assessment instruments but also the absence of a shared conceptual framework defining the core IPC competencies expected of undergraduate nursing students. Consequently, differences in reported outcomes may reflect variations in measurement approaches as much as true differences in students’ competencies.

These findings highlight the need for greater standardisation of assessment methods in undergraduate nursing education. Future research should prioritise the development and validation of comprehensive IPC assessment instruments with robust psychometric properties and clearly defined conceptual frameworks. Greater methodological consistency would facilitate comparisons across educational settings, strengthen the evidence base for infection prevention education, and support the evaluation of educational interventions aimed at improving nursing students’ IPC competencies, in line with the educational principles underpinning the World Health Organization Core Components of Infection Prevention and Control Programmes [65].

### 4.6. Strengths and Limitations

This review has several strengths. To our knowledge, it is the first systematic review to synthesise infection prevention and control (IPC) competencies among nursing students using a construct-based approach, integrating evidence across knowledge, attitudes, perceptions, self-reported practices, adherence, compliance, and assessment instruments. In addition, it provides a comprehensive overview of the instruments used to assess IPC competencies, highlighting important methodological heterogeneity in both assessment methods and the interpretation of knowledge.

Several limitations should be acknowledged. The original search strategy was designed to identify studies evaluating knowledge, in line with the primary objective of the review. However, because the review also narratively synthesised related constructs such as attitudes, perceptions, self-reported practices, adherence, and compliance, studies focused exclusively on these outcomes and not indexed with knowledge-related terms may have been missed. Therefore, the findings regarding attitudes, practices, adherence, perceptions, and compliance should be interpreted as complementary rather than exhaustive. In addition, the review protocol was not prospectively registered in PROSPERO, and only studies published in English and Spanish were included, which may have introduced language bias. Furthermore, the methodological heterogeneity of the included studies, together with the predominance of cross-sectional designs and self-reported measures, limited direct comparisons and precluded quantitative synthesis. Nevertheless, the consistency of the main findings across different educational settings supports the robustness of the conclusions.

## 5. Conclusions

This systematic review provides a comprehensive synthesis of the current evidence on undergraduate nursing students’ knowledge of infection prevention and control (IPC) related to HH, SPs, and broader IPC recommendations. The findings indicate that nursing students generally demonstrate satisfactory levels of IPC knowledge, although important gaps remain in specific areas. Higher knowledge levels were associated with factors such as advanced academic progression, previous IPC training, and greater clinical exposure, although findings were not entirely consistent across studies.

Beyond knowledge, the included studies suggest that attitudes, perceptions, self-reported practices, adherence, and compliance may influence the application of IPC principles in clinical settings. These findings support the importance of educational approaches that combine knowledge acquisition with opportunities for practical application and reinforcement during clinical placements.

Finally, considerable heterogeneity was identified in the instruments used to assess IPC knowledge and related outcomes. The development and adoption of standardised, psychometrically robust assessment tools and greater consensus regarding core IPC domains would enhance comparability across studies, strengthen the evaluation of educational interventions, and support improvements in infection prevention education for future nurses.

## Figures and Tables

**Table 2:** Validated and ad hoc assessment instruments used to evaluate infection prevention competencies among nursing students.

Instrument	Domain	Main Construct(s) Assessed	Validation
WHO Five Moments-based questionnaires	HH	Knowledge/Self-reported practices	WHO framework
HHQ	HH	Knowledge, attitudes	Validated
HHPI	HH	Self-reported practices	Validated
HHBS	HH	Beliefs	Validated
CSPS	SP	Compliance	Validated
FIASPS-SV	SP	Determinants of adherence	Validated
QKSP	SP	Knowledge of standard precautions	Validated
QCSP	SP	Compliance with standard precautions	Validated
ICSQ	IPC	Infection prevention knowledge	Validated
LCQ-IP	IPC	Organisational climate/perceptions	Validated
Ad hoc questionnaires	Mixed	Mixed constructs	Non-validated (some adapted from previously published instruments)

## Data Availability

All data related to this systematic review are included in the published article.

## Abbreviations

The following abbreviations are used in this manuscript:

ABHS	Alcohol-based handrubs
CDC	United States Centers for Disease Control and Prevention
CINAHL	Cumulative Index to Nursing and Allied Health Literature
CSPS	Compliance with Standard Precautions Scale
ECDC	European Centre for Disease Prevention and Control
COVID-19	Coronavirus disease 2019
EMBASE	Excerpta Medica Database
FIASPS-SV	Factors Influencing Adherence to Standard Precautions Scale–Student Version
HAI	healthcare-associated infection
HH	hand hygiene
HHQ	The Hand Hygiene Questionnaire
ICSQ	Infection Control Standardized Questionnaire
IPC	Infection Prevention and Control
JBI	Joanna Briggs Institute
LCQ-IP	Leading a Culture of Quality for Infection Prevention
MEDLINE	Medical Literature Analysis and Retrieval System Online
NS	Nursing Student
PEO	Population/Exposure/Outcome
PPE	Personal Protective Equipment
PRISMA	Preferred Reporting Items for Systematic Reviews and Meta-Analyses
SARS-CoV-2	Severe Acute Respiratory Syndrome Coronavirus 2
SPs	Standard Precautions
WHO	World Health Organization
WOS	Web of Science
5MHH	Five Moments for Hand Hygiene

## Appendix A

**Table A1:** Database-specific search strategies.

Database	Search Strategy	Filters and Limits	Final Search Date
MEDLINE via PubMed	(“Students, Nursing”[MeSH Terms] OR “Students, Health Occupations”[MeSH Terms] OR “Education, Nursing”[MeSH Terms] OR “Education, Nursing, Baccalaureate”[MeSH Terms] OR “Schools, Nursing”[MeSH Terms] OR (nurs*[tw] AND student*[tw]) OR (nurs*[All Fields] AND student*[All Fields]) OR “nursing student*”[tw] OR “nursing student*”[All Fields] OR “student nurse*”[tw] OR “student nurse*”[All Fields] OR “nurse student*”[tw]) AND (“Hand Hygiene”[MeSH Terms] OR “Hand Disinfection”[MeSH Terms] OR “Infection Control”[MeSH Terms] OR “Universal Precautions”[MeSH Terms] OR “Personal Protective Equipment”[MeSH Terms] OR “Cross Infection”[MeSH Terms] OR “hand hygien*”[tw] OR handwashing[tw] OR “hand washing”[tw] OR “hand disinfection”[tw] OR “standard precaution*”[tw] OR “universal precaution*”[tw] OR “infection prevention and control”[tw] OR “infection prevention”[tw] OR “infection control”[tw] OR “nosocomial infection*”[tw] OR “healthcare-associated infection*”[tw] OR “healthcare associated infection*”[tw] OR “health care associated infection*”[tw] OR “hospital-acquired infection*”[tw] OR “hospital acquired infection*”[tw] OR HAI[tw] OR HAIs[tw] OR antisepsis[tw] OR asepsis[tw] OR “aseptic technique*”[tw] OR “personal protective equipment”[tw] OR PPE[tw] OR glove*[tw] OR mask*[tw] OR gown*[tw] OR goggle*[tw]) AND (“Health Knowledge, Attitudes, Practice”[MeSH Terms] OR knowledge[tiab])	English or Spanish; no date restriction	[7 January 2026]
CINAHL via EBSCOhost	(MH “Students, Nursing+” OR TX “nursing student” OR TX “nursing students” OR TX “nurse student” OR TX “nurse students” OR TX “student nurse” OR TX “student nurses” OR TX (nurs* N1 student*)) AND (MH “Hand Hygiene” OR MH “Infection Control+” OR MH “Universal Precautions” OR MH “Cross Infection+” OR MH “Protective Devices+” OR TX “hand hygiene” OR TX “standard precaution” OR TX “standard precautions” OR TX “infection prevention and control” OR TX “infection prevention” OR TX “nosocomial infection” OR TX “nosocomial infections” OR TX “healthcare-associated infection” OR TX “healthcare-associated infections” OR TX “health care-associated infection” OR TX “health care-associated infections” OR TX antisepsis OR TX “personal protective equipment” OR TX PPE OR TX glove* OR TX mask* OR TX gown* OR TX goggle*) AND (MH “Knowledge+” OR TX knowledge)	English or Spanish; no date restriction	[7 January 2026]
Cochrane Library	((nursing OR nurse OR nurses OR nurs*) AND (student OR students OR undergraduate OR undergraduates OR student*)) AND (“hand hygiene” OR handwashing OR “hand washing” OR “hand disinfection” OR “standard precaution” OR “standard precautions” OR “universal precaution” OR “universal precautions” OR “infection prevention and control” OR “infection prevention” OR “infection control” OR “infection prevention measures” OR “nosocomial infection” OR “nosocomial infections” OR “healthcare associated infection” OR “healthcare associated infections” OR “healthcare-associated infection” OR “healthcare-associated infections” OR “health care associated infection” OR “health care associated infections” OR “health care-associated infection” OR “health care-associated infections” OR “hospital acquired infection” OR “hospital acquired infections” OR “hospital-acquired infection” OR “hospital-acquired infections” OR antisepsis OR asepsis OR “aseptic technique” OR “aseptic techniques” OR “personal protective equipment” OR PPE OR glove OR gloves OR mask OR masks OR gown OR gowns OR goggle OR goggles) AND knowledge	English or Spanish where available; no date restriction	[7 January 2026]
Embase	(“nursing student” OR “nursing student” OR “nursing students” OR “nursing students” OR “student nurse” OR “student nurse” OR “student nurses” OR “student nurses”) AND (“hand hygiene” OR “hand hygiene” OR “standard precaution” OR “standard precaution” OR “standard precautions” OR “standard precautions” OR “infection prevention and control” OR “infection prevention and control” OR “infection prevention” OR “infection prevention” OR “infection control” OR “infection control” OR “nosocomial infection” OR “nosocomial infection” OR “nosocomial infections” OR “nosocomial infections” OR “healthcare-associated infection” OR “healthcare-associated infection” OR “healthcare-associated infections” OR “healthcare-associated infections” OR “health care-associated infection” OR “health care-associated infection” OR “health care-associated infections” OR “health care-associated infections” OR antisepsis OR antisepsis OR “personal protective equipment” OR “personal protective equipment” OR PPE OR PPE OR glove OR glove OR gloves OR gloves OR mask OR mask OR masks OR masks OR gown OR gown OR gowns OR gowns OR goggle OR goggle OR goggles OR goggles) AND (knowledge OR knowledge)	English or Spanish; no date restriction	[7 January 2026]
Scopus	TITLE-ABS-KEY ((“nursing student” OR “nursing students”) AND (“hand hygiene” OR “standard precaution” OR “standard precautions” OR “infection control” OR “nosocomial infection” OR “nosocomial infections” OR antisepsis OR “personal protective equipment” OR glove OR gloves OR mask OR masks OR gown OR gowns OR goggle OR goggles) AND (knowledge))	English or Spanish; article document type if applied; no date restriction	[7 January 2026]
Web of Science	TS = (((nurs* student* OR nursing student*) AND (hand hygiene OR standard precaution* OR infection prevention and control OR infection prevention OR nosocomial infection* OR healthcare-associated infection* OR antisepsis OR personal protective equipment OR PPE OR glove* OR mask* OR gown* OR goggle*) AND knowledge)	English or Spanish; article document type if applied; no date restriction	[7 January 2026]

## Appendix B

**Table A2:** Hand Hygiene (HH).

Study	Country	Participant Characteristics and Comparison Groups	Instrument or Model Used	Main Description of Results
(Jeong & Kim, 2016) [15]	South Korea	*N* = 208 NSS in third- or fourth-year of nursing school; mean age: 21.99 ± 1.71 years; 88.9% female.	A questionnaire about HH knowledge, beliefs (behaviour, normative, and control beliefs), and behaviour	Behavioural beliefs were positively correlated with normative beliefs and HH behaviour, while control beliefs also correlated with HH behaviour. HH knowledge scores were moderate (68.1% correct responses). The highest-scoring item concerned prevention of microbial colonization through skin care (99.5%), whereas the lowest-scoring item related to replacing HH with glove use (10.6%).
(Bounou et al., 2021) [14]	Greece	*N* = 132 MSs and 111 NSs (all the students were in their final year); mean age: 21.8 years NS; 80% NSs were female.	32 items based on the 5MHH (including 22 closed questions): (1) self-reported HH practices; (2) HH knowledge; (3) attitudes on the effectiveness of HH in controlling HAIs and their knowledge and practices; (4) attitudes on HH education; and (5) self-reported HH education type and level.	Medical and nursing students differed significantly in several aspects of HH education and training. Nursing students reported greater exposure to compulsory HH education during preclinical years and greater awareness of hospital HH supervision, whereas medical students more frequently received theoretical training and were more influenced by trainers’ behaviour. Infection risk was the main factor influencing HH attitudes in both groups. Overall, medical students reported higher HH implementation levels than nursing students.
(Kingston et al., 2018) [27]	Ireland	*N* = 225 NSs and 98 MSs; mean age unspecified; 94% NSs were female.	The WHO 5MHH	Nursing students showed greater compliance with the 5MHH model than medical students, particularly after exposure to bodily fluids, whereas compliance was lowest after contact with patient surroundings in both groups. Nursing students also demonstrated greater awareness of the clinical relevance of HH, perceived benefits for patient outcomes, and adherence to HH recommendations. However, medical students reported more frequent use of alcohol-based hand sanitiser.
(Oyapero & Oyapero, 2018) [53]	Nigeria	*N* = 69 NSs: first- (14), second- (31), and third-year (24); mean age: 20.8 ± 2.44 years; 95.7% female.	(1) self-administered ad hoc questionnaire measuring sociodemographic characteristics; (2) a modified version of the 5MHH.	Positive health beliefs were mainly associated with acting as an HH role model, supervisor reinforcement, and inclusion of HH in the curriculum, whereas negative beliefs were linked to observing poor practices among senior staff. HH compliance was highest during wound care procedures, and demonstration and clinical practice were considered the most effective teaching methods. Third-year nursing students showed better HH behaviour.
(Cambil-Martin et al., 2020) [23]	Spain	*N* = 303 NSs and 657 MSs (second- or third-years); mean age: 21.91 ± 3.15 years NS; 76.1% NSs were female.	Analysis of HH behaviour before and after patient contact; comparison of the knowledge and attitudes of MSs and NSs towards HH using the 5MHH.	Mean HH knowledge scores were high, with most students scoring above 4 out of 6. Nursing students achieved significantly higher scores than medical students across all assessed dimensions, and prior HH education or training was associated with better knowledge scores.
(Kacan, 2021) [58]	Turkey	*N* = 305 NSs (all years); mean age unspecified; 81.0% female.	The Hand Hygiene Practices Inventory (HHPI) adapted to Turkish by Van de Mortel [49].	Students who considered HH “very important” and female students demonstrated significantly higher HHPI scores. HH compliance was highest after patient contact and lowest before glove use.
(Birgili & Ugurlu, 2019) [67]	Turkey	*N* = 655 NSs; mean age: 21.44 ± 1.94 years; 64.9% female.	The Hand Hygiene Practices Inventory (HHPI) adapted to Turkish by Van de Mortel [49].	Overall HH scores were high across all assessed dimensions. Female students achieved significantly higher HHPI, HIS (Hand Hygiene Importance Scale), and total HHQ scores, while significant differences were also observed across age groups and academic years. Fourth-year students showed the highest HHPI and overall HHQ scores.
(Bargellini et al., 2014) [21]	Italy	*N* = 100 NSs and 100 MSs from all academic years; mean age: 24.2 ± 5.4 years NS, respectively; 76% NSs were female.	An ad hoc instrument to assess HH procedures (hand washing and/or hand rubbing) and use of PPE such as gloves, goggles, or masks.	All nursing students reported performing at least one HH practice, with significantly higher frequencies of handwashing, hand rubbing, and glove use than medical students. Nursing students also more frequently recognised HH as a self-protection measure and reported receiving adequate theoretical HH instruction.
(Maras et al., 2024) [57]	Turkey	*N* = 330 NSs first-, second-, and third-year; mean age: 19.80 ± 1.30 years; 76.1% female.	The 5MHH; NSs were also asked their opinions and attitudes regarding the HH behaviours of healthcare providers.	Prior education on HAIs was significantly associated with recognising HH as an important component of inpatient care. Approximately half of the students had received HAI education, mainly through healthcare professionals during academic training.
(Singh & Barnard, 2023) [68]	South Africa	*N* = 51NSs, 413 other health sciences students (MS, Optometry, Podiatry, Chiropractic, …); mean age all health sciences 23 years; 77.4% all health sciences were female.	Ad hoc instrument about handwashing frequency, knowledge, practices, and skills.	The main reasons for skipping handwashing were substituting it with ABHS use and forgetting to wash hands. A small proportion of students believed glove use eliminated the need for handwashing. Female and nursing students demonstrated higher HH knowledge scores overall.
(Skodova et al., 2014) [44]	Spain	*N* = 143 NSs and 403 MSs second-, and third-year; mean age: 21.2 ± 4.77 years NS; 68.5% NSs were female.	The distribution of a fluorescent hydroalcoholic solution (HAS) was visually assessed on the palms, back of the hand, between the fingers, and finger tips/nails, for both hands using an ultraviolet lamp.	Palms showed the best HAS coverage, whereas thumbs and interdigital spaces were the most frequently missed areas. Approximately half of the students achieved good or very good coverage, while male first-year nursing students demonstrated the poorest performance. Inadequate sanitisation was most common on the backs of the hands, thumbs, and between the fingers.
(Thakker & Jadhav, 2015) [37]	India	*N* = 40 NSs, 84 MSs, 74 Dental Students in second year; mean age unspecified; no % female.	Evaluate and compare HH knowledge in MSs, Dental Students, and NSs using the 5MHH, considering scores > 75% as good, 50–74% moderate, and <50% poor.	Knowledge of HH transmission routes and WHO recommendations was limited, particularly regarding the main sources of healthcare-associated infections and recommended ABHS duration. Overall, only 7.6% of students demonstrated good HH knowledge, with medical students outperforming dental and nursing students.
(Nair et al., 2014) [38]	India	*N* = 46 NSs and 98 MSs; mean age unspecified; no % female.	HH knowledge was assessed using the 5MHH and attitudes and practices were assessed using another self-structured questionnaire, 10 and 6 questions, respectively. Respondents were given the option to select on a 1- to 7-point scale between strongly agree and strongly disagree. maximum score for attitude is 10 and for practice it is 6 considering scores > 75% as good, 50–74% moderate, and <50% poor.	Overall HH knowledge was low, with only 9% of students demonstrating good knowledge. Nursing students showed significantly better HH knowledge, attitudes, and practices than medical students, likely due to higher rates of formal HH training.
(Saurabh et al., 2022) [66]	India	*N* = 50 NSs and 160 MSs in last year bachelor’s degree students; mean age unspecified; 64.8% NSs were female.	A self-administered questionnaire based on the CDC HH guidelines	Over half of the participants demonstrated good HH knowledge, although many students incorrectly believed that glove use reduced the need for HH. Despite most students recognising HH as an essential professional responsibility, forgetting HH practices and time constraints were commonly reported barriers.
(Oh, 2021) [43]	Korea	*N* = 233 NSs third- and fourth-year; mean age 22.43 ± 1.53 years; 82.0% female.	Modified 5MHH to assess participant knowledge and perceptions of HH.	Study year, university-affiliated hospital training, and recent clinical practice courses significantly influenced HH perceptions. HH knowledge was positively associated with HH performance, which was also influenced by healthcare workers’ HH behaviour. Final-year and male students demonstrated higher HH perception scores.
(Khan et al., 2021) [16]	Pakistan	*N* = 57 NSs, 60 MBBS, 48 health sciences from different bachelor’s degree courses; mean age: 21.36 ± 1.53 years NS; 81.8% NSs were female.	Questionnaire based on 5MHH for prevention of HAIs: knowledge, practices, and attitudes.	Significant differences in needle-recapping practices were observed across student groups, with nursing students demonstrating the highest levels of correct responses, knowledge, and practice scores compared with medical and allied health sciences students.
(Hung et al., 2017) [61]	Hong Kong	*N* = 421 NSs first-, second-, and third-year; mean age unspecified; no % female.	A validated, self-reported HH questionnaire (HHQ) developed by van de Mortel [49].	HH knowledge and performance improved significantly across study years, with higher scores observed among more advanced students.
(Cruz & Bashtawi, 2016) [25]	Saudi Arabia	*N* = 198 NSs second- to fourth-year; mean age: 21.77 ± 1.26 years; 46% female.	A self-administered ad hoc HH questionnaire based on the 5MHH, and the Attitude Scale and Practice Scale (Chauhan, s. f.; Singh & Barnard, 2023 [68]) to identify the predictors of HH practice among Saudi Arabian NSs.	Most students had attended HH training within the previous six months, and overall HH knowledge was moderate. Positive attitudes toward HH were the strongest predictor of HH behaviour, followed by awareness of HH effectiveness and participation in HH training.
(Kingston et al., 2017) [26]	Ireland	*N* = 225 NSs first- to fourth-year; mean age unspecified; 94% female.	5MHH including HH and a section specifically addressing hand rubbing using ABHS.	A small proportion of students were unable to distinguish when to use soap and water versus ABHS. Negative perceptions of ABHS and skin-related problems were common barriers to its use. HH compliance was lower before and after patient contact.
(Sundal et al., 2017) [69]	Norway	*N* = 29 NSs second-year; aged 21–48 years; 93.1% female.	A standardised and validated HH observation tool based on the 5MHH	Compliance was highest after touching patient surroundings, after patient contact, and following exposure to bodily fluids, whereas lower compliance was observed before patient contact and prior to clean or aseptic procedures. Nursing staff demonstrated significantly lower compliance before than after patient contact.
(Hernandez-Garcia & Cardoso, 2013) [45]	Spain	*N* = 146 NSs from different years of their degree; mean age unspecified; no % female.	A questionnaire comprising 12 situations requiring HH, examining compliance on a 5-point Likert scale. HH knowledge was also measured via 13 multiple-choice questions.	Approximately half of the nursing students across academic years met HH standards. Female sex and greater knowledge of HAIs and appropriate ABHS use were significant predictors of acceptable HH compliance.
(Ceylan et al., 2020) [62]	Turkey	*N* = 563 NSs from different course years; mean age: 21.51 years; 87% female.	Instrument evaluating HH practices using a Hand Hygiene Beliefs Scale (HHBS), Hand Hygiene Practices Inventory (HHPI), and “Derma LiteCheck”.	Female nursing students reported significantly higher handwashing frequency than males. HH was most frequently performed after toilet use and after contact with blood or bodily fluids. Cleanliness was highest on the palms and backs of the hands, whereas the nails showed the poorest sanitisation results.
(Syed & Al-Rawi, 2024) [70]	Saudi Arabia	*N* = 304 NSs from first-, second- and third-year; mean age: 21.59 ± 1.93 years; 33.9% female.	The questionnaire consisted of three sections: (1) demographic variables; (2) hand-washing knowledge items; and (3) hand hygiene practice items assessed using a three-point Likert-type scale.	Most students reported performing HH before and after patient contact, after exposure to body secretions, and before aseptic procedures. Soap use was more common than alcohol-based hand rub use. Female students demonstrated significantly higher HH knowledge and practice scores, whereas no differences were observed according to age, study year, or marital status.
(Blomgren et al., 2024) [64]	Sweden	*N* = 117 NSs in their first semester (NSS1 [*n* = 71]), the last semester (NSS6 [*n* = 46]); mean age 25.5 years; 82.05% female.	The questionnaire based on the 5MHH	Senior nursing students demonstrated significantly higher HH knowledge scores than first-year students. Both groups showed limited understanding of patients as the main source of microorganisms and of HH practices after exposure to bodily fluids and patient surroundings. First-year students showed poorer knowledge regarding HH before aseptic procedures and appropriate HH methods in clinical situations.
(Linnik et al., 2024) [28]	Europe (Spain, Turkey, Estonia, Portugal and Finland)	*N* = 638 NSs (35.7% Spain, 26.2% Turkey, 17.1% Estonia, 10.7% Portugal, 10.3% Finland); mean age 21.7 years; 83.2% female.	The questionnaire consisted of three sections: (1) demographic variables; (2) 5MHH; (3) evaluated their general self-efficacy and self-efficacy related to infection prevention control	Significant differences in HH knowledge and self-efficacy were observed between countries. Finnish nursing students demonstrated the highest HH knowledge scores, whereas Estonian students reported higher general and infection-control-related self-efficacy. Knowledge deficits were mainly related to sources of healthcare-associated pathogens and appropriate HH methods in clinical situations.
Ntaote et al., 2024 [56]	Lesotho	*N* = 103 NSs from second- to fourth-year; mean age: 22.3 ± 2.4 years; 88% female.	Self-administered questionnaire adapted from the WHO Hand Hygiene Monitoring Tools and Mbroh et al., assessing HH knowledge, attitudes and perceptions.	Most nursing students demonstrated moderate HH knowledge, positive attitudes, and favourable perceptions towards hand hygiene. Students with greater HH knowledge also reported more positive attitudes and perceptions. Knowledge deficits were mainly related to the WHO 5 Moments for Hand Hygiene and appropriate use of alcohol-based hand rub.

**Table A3:** Standard Precautions (SP).

Study	Country	Participant Characteristics and Comparison Groups	Instrument or Model Used	Main Description of Results
(Khan et al., 2021) [16]	Saudi Arabia	*N* = 81 NSs, 154 MSs, 255 Dental Students, 68 Pharmacy Students, 48 Physiotherapy students; mean age unspecified; 13.4% NSs were female.	WHO questions about the use and knowledge of PPE.	PPE use differed significantly across clinical specialties. Face masks were the most commonly used PPE, whereas shoe covers were the least used. Knowledge of OSHA guidelines and correct donning and doffing procedures was limited, and only a small proportion of nursing students considered PPE training important.
(Bouchoucha et al., 2021) [22]	Australia	*N* = 321 NSs; mean age: 26 years; 91% female.	CSPS and FIASPS-SV.	CSPS scores indicated moderate-to-high compliance across standard precaution domains, with lower scores for spill and equipment contamination management. Leadership scores were higher among male students, those with prior healthcare experience, and third-year students. Contextual cues and leadership were identified as the main determinants of adherence to standard precautions, whereas higher justification scores were associated with lower compliance across multiple domains.
(Ayele et al., 2022) [41]	Ethiopia	*N* = 414 NSs from third- and fourth-year; mean age: 24; 46.6% female.	CSPS, 3 questions regarding factors affecting compliance in NSs.	Just over half of the nursing students complied with SPs, while good knowledge and positive attitudes were less common. Most students perceived the workplace climate as unsafe. Better SP compliance was significantly associated with greater SP knowledge, recent SP training, and safer workplace climate perceptions.
(Alshammari et al., 2018) [20]	Saudi Arabia	*N* = 829 NSs four-year programme; mean age: 22.26; 69.5% female.	CSPS.	Compliance was highest for correct mask use and lowest for appropriate sharps container disposal. Significant differences in SP compliance were observed across universities and academic years, while recent IPC training was associated with higher compliance.
(van der Berg & Daniels, 2013) [40]	South Africa	*N* = 253 NSs in their second to the fourth year of study; mean age unspecified; 83% female.	Self-administered instrument to examine participant knowledge of Universal Precautions (Ups).	Knowledge and practice of UPs showed a positive but non-significant correlation. Students demonstrated mixed understanding of UPs, particularly regarding isolation requirements, occupational exposure risks, and situations in which UPs are unnecessary. Most students correctly identified that used needles should not be recapped.
(van Gulik et al., 2021) [29]	Thailand	*N* = 533 fourth-year NSs; aged 20–24 years; 95.7% female.	CSPS and FIASPS-SV.	Self-reported compliance with SPs was highest for sharps disposal and lowest for decontamination of spills and contaminated equipment. Fourth-year nursing students demonstrated significantly better cross-infection prevention practices than third-year students.
(de Lourdes Lopes et al., 2023) [63]	Brazil	*N* = 161 NSs in the fourth academic year; aged 18–28 years; 90.7% female.	Questionnaire on Knowledge on Standard Precautions (QKSP) and Questionnaire for Compliance with Standard Precautions (QCSP).	Nursing students demonstrated satisfactory SP knowledge and compliance overall. Significant differences in SP compliance were observed between advanced academic periods. Most students reported proper sharps disposal, although some did not appropriately manage contaminated sharps injuries.
(AL-Rawajfah & Tubaishat, 2015) [39]	Jordan	*N* = 583 NSs third-year; mean age: 21.2 ± 2.6 years; 65.3% female.	A tool developed by Chan et al. [71] about SP knowledge and practices.	Most students achieved high knowledge and practice scores. Previous IPC education was associated with better knowledge and practice outcomes.
(Cheung et al., 2015) [54]	Hong Kong	*N* = 678 NSs second- to fourth-year; mean age: 21.02 ± 1.46 years; sex distribution: 3:1 female/male.	Ad hoc instrument about needlestick injuries and the source of information on SPs.	Second- and fourth-year nursing students demonstrated better SP compliance than third-year students. Perceived influence of nursing staff significantly affected compliance, particularly among second- and third-year students. Main sources of SP information were university teachers and clinical staff. Key predictors of SP compliance included SP knowledge, perceived barriers, adequacy of training, management support, and nursing staff influence.
(Coşkun Palaz & Demir Erbaş, 2025) [59]	Turkey	*N* = 405 NSs second- to fourth-year; mean age: 21.43 ± 1.68 years; 79.8% female.	Questionnaires: Nurses’ Infection Prevention and Control Observation Questionnaire, Compliance with Standard Precautions Scale (CSPS) and The Factors Influencing Adherence to Standard Precautions Scale-Student version (FIASPS-SV)	Most students considered their IPC knowledge sufficient, although barriers to SP compliance included lack of materials, rushing, anxiety, indifference, and limited knowledge. SP compliance was significantly associated with academic year and IPC knowledge, with second-year students showing higher compliance scores than fourth-year students. Perceived leadership and action cues were positively associated with SP compliance, whereas justification showed a weak negative association.
(Erwin et al., 2026) [51]	Indonesia	*N* = 242 NSs in professional clinical training; mean age: 28.48 ± 7.37 years; 83.1% female.	Cross-sectional questionnaire based on the Theory of Planned Behaviour (TPB), assessing perceived behavioural control (PBC), knowledge of nosocomial infections and PPE use, and behavioural intention.	High perceived behavioural control and behavioural intention scores were observed among nursing students. Staff direction and specific training showed the strongest univariate associations with intention to maintain a safe clinical environment. However, in the multivariate model, knowledge of PPE use was the only significant independent predictor of intention (AOR = 2.66, *p* = 0.003), highlighting the importance of technical infection-prevention knowledge.

**Table A4:** Infection prevention and control (IPC).

Study	Country	Participant Characteristics	Instrument or Model Used	Main Description of Results
(Majidipour et al., 2019) [42]	Iran	*N* = 102 NSs from third- and fourth-year; mean age: 23.83 ± 1.81 years; 48% female.	Ad hoc questionnaire with 18 items on knowledge of the standards of HAI control.	Female nursing students and third-year students demonstrated significantly better performance. Knowledge of HAI control showed a moderate positive correlation with performance standards.
(Tumala et al., 2019) [55]	Saudi Arabia	*N* = 829 NSs studying at 6 Saudi Arabian universities; mean age unspecified; 69.5% female.	The LCQ-IP was used.	The mean LCQ-IP score reflected a modest perception of the hospital IPC training climate. Quality prioritisation and improvement orientation received the highest ratings, whereas psychological safety and supportive environment scored lowest. Perceptions of the IPC climate differed significantly across universities.
(Mitchell et al., 2014) [50]	Australia	*N* = 349 NSs from six universities; mean age: 25 years; 60.4% female.	An online survey was developed with the help of IPC experts and cross-referenced against national guidelines.	Students demonstrated significantly better knowledge of standard precautions than of transmission-based precautions. Self-reported IPC compliance was not associated with sex or age. IPC professionals and hospital policies were the main information sources, whereas younger students more frequently consulted senior nurses and the internet.
(Kim & Park, 2021) [52]	South Korea	*N* = 178 NSs; 82.02% students from all academic years; mean age: 22.75 ± 2.28 years. No % female.	Self-administered survey assessing knowledge and attitudes toward IPC, perception of the existence of a safe environment in terms of IPC, and compliance with IPC during student clinical practice.	Most students had received IPC training, although many reported needing additional education on SPs following infection exposure. Needlestick injuries and exposure to blood or bodily fluids were associated with lower compliance in cross-infection prevention, PPE use, and decontamination practices. Perceptions of a safe environment and positive IPC attitudes were significant predictors of IPC compliance.
(Choi & Kim, 2018) [24]	Korea	*N* = 350 NSs with more than 1 year of clinical experience; mean age unspecified; 89.1% female.	Ad hoc questions about non-compliance with ICP by staff nurses and the NSs’ intention to comply with ICP.	Nursing students’ intention to comply with ICP was positively associated with awareness of infection control practices and training in larger hospitals, whereas observing non-compliance among nursing professionals negatively influenced their intention to comply.
(Alriyami et al., 2022) [19]	Oman	*N* = 330 NSs from all academic years; mean age: 21.57 ± 1.9 years; 56.4% female.	The ICSQ.	Female nursing students achieved significantly higher mean scores than males. Students relying primarily on clinical experience differed significantly from those using the curriculum or internet as their main knowledge sources.
(Ratcliffe & Smith, 2014) [72]	England	*N* = 89 NSs from third-year; mean age unspecified; 89% female.	Ad hoc data from a survey about knowledge, skills, decision-making strategies, and wider contextual factors influencing NS practice.	Most nursing students were aware of their placement site’s IPC policy, although fewer had read it or attended IPC training sessions. Students generally demonstrated good knowledge regarding situations requiring glove use during patient care procedures.

**Table A5:** Miscellaneous.

Study	Country	Participant Characteristics	Instrument or Model Used	Main Description of Results
(Zimmerman et al., 2020) [49]	Australia	*N* = 225 NSs from different bachelor’s nursing degree courses; aged 18–25 years; 93.7% female.	Ad hoc survey about IPC knowledge and the variables that influenced its practice.	HH knowledge was significantly associated with year of nursing study, particularly regarding infection chain links, glove use, management of needlestick injuries, and sharps-injury prevention measures.
(Baba et al., 2023) [46]	Morocco	*N* = 159 NSs; mean age: 20.74 ± 1.04 years; 73.5% female.	Ad hoc survey about knowledge of HAIs and attitudes and practices for their prevention.	Most nursing students demonstrated unsatisfactory general knowledge of HAI prevention. General nursing students achieved better knowledge, attitudes, and practices scores than students in emergency, intensive care, or mental health specialties. Although most students recognised that HAIs are transmissible, knowledge of HAI hosts and reservoirs remained limited.
(Khubrani et al., 2018) [35]	Saudi Arabia	*N* = 14 NSs (third- and fourth-year), 75 MSs, 18 Dental Students, 17 Medical Sciences, 5 Pharmacy (from different courses). 80.6% were female (all health sciences).	An ad hoc survey with 41 items measuring NS knowledge of SPs and IPC.	Most nursing students demonstrated sufficient IPC and SP knowledge, with female students achieving higher scores than males. The highest-scoring domains were general SP concepts, HH, and PPE use, whereas knowledge related to sharps disposal and healthcare provider care was lower. Nursing students demonstrated higher knowledge levels than students from other healthcare disciplines.
(Darawad & Al-Hussami, 2013) [60]	Jordan	*N* = 114 NSs from all academic years; mean age: 21.37 years; 76% female (*n* = 87).	An ad hoc survey on IPC knowledge and attitudes and compliance with universal IPCs.	Nursing students demonstrated inadequate IPC knowledge, positive attitudes, and moderate compliance. Positive IPC attitudes were the strongest predictor of compliance and showed a significant positive correlation with both compliance and IPC knowledge.
(Brosio et al., 2017) [47]	Italy	*N* = 339 NSs (first- to third-years); mean age: <22 years (64.3%), ≥22 years (35.7%); 74.6% female.	An ad hoc survey about HAI practices, SPs, and HH.	Most nursing students correctly identified major HAI risk factors, although knowledge of environmental sources of HAIs improved significantly with academic progression. Knowledge regarding glove use and substitution of handwashing with alcohol-based hand sanitizer remained limited across all academic years. Overall knowledge was adequate, but HAI- and HH-specific knowledge remained insufficient, even among final-year students.
(Ojulong et al., 2013) [36]	Namibia	*N* = 114 NSs, 31 MSs, and 17 radiography students (RSs), all in their second- or third-years.; mean age: 24.3 ± 0.8; 75.3% female.	IPC	Medical students achieved higher overall HAI knowledge scores than nursing and radiography students. Knowledge deficits were common regarding environmental HAI reservoirs, appropriate ABHS use, and glove use according to standard precautions.
(Sharma & Bachani, 2023) [7]	India	*N* = 177 NSs, 423 MSs; mean age unspecified; 73% NSs were female.	Questions based on CDC and WHO guidelines: knowledge and attitudes toward SP, post-exposure prophylaxis (PEP), HAIs, and self-reported practice and barriers to IPC, SPs, and PEP compliance.	Knowledge of PEP, PPE, hazard symbols, and correct HH implementation was limited among both medical and nursing students, despite generally high theoretical HH knowledge. Misconceptions regarding ABHS use on visibly soiled hands and low awareness of the WHO 5 Moments for Hand Hygiene were common. High workload and limited knowledge were identified as major barriers to SP compliance.
(Yıldız et al., 2024) [48]	Cyprus	*N* = 122 NSs third- and fourth-year; mean age 22.55 ± 1.53 years; 68% female.	PPE and Surgical Hand-Washing (SHW) information form”	Nursing students demonstrated high accuracy in several PPE use and SHW items, although overall knowledge levels were moderate. Third-year students achieved significantly higher PPE scores than fourth-year students, while female students generally obtained higher scores across domains.

## Appendix C

**Table A6:** Critical Appraisal of Eligible Analytical Cross-Sectional Study (JBI).

Citation	Q1	Q2	Q3	Q4	Q5	Q6	Q7	Q8	Total Yes	%
(Oyapero & Oyapero, 2018) [53]	Y	Y	Y	Y	N	N	Y	Y	6/8	75
(Bouchoucha et al., 2021) [22]	Y	Y	Y	Y	Y	Y	Y	Y	8/8	100
(Alriyami et al., 2022) [19]	Y	Y	Y	Y	Y	Y	Y	Y	8/8	100
(Tumala et al., 2019) [55]	Y	Y	Y	Y	Y	Y	Y	Y	8/8	100
(Mitchell et al., 2014) [50]	Y	Y	Y	Y	Y	Y	Y	Y	8/8	100
(Cambil-Martin et al., 2020) [23]	Y	Y	Y	Y	Y	U	Y	Y	7/8	87.5
(Kim & Park, 2021) [52]	Y	Y	Y	Y	Y	Y	Y	Y	8/8	100
(Ayele et al., 2022) [41]	Y	Y	Y	Y	Y	Y	Y	Y	8/8	100
(Alshammari et al., 2018) [20]	Y	Y	Y	Y	Y	Y	Y	Y	8/8	100
(van der Berg & Daniels, 2013) [40]	Y	Y	Y	Y	Y	Y	Y	Y	8/8	100
(Bounou et al., 2021) [14]	Y	Y	Y	Y	U	U	Y	Y	6/8	75
(Kacan, 2021) [58]	Y	Y	Y	Y	Y	Y	Y	Y	8/8	100
(Birgili & Ugurlu, 2019) [67]	Y	Y	Y	Y	Y	Y	Y	Y	8/8	100
(Ceylan et al., 2020) [62]	Y	Y	Y	Y	Y	Y	Y	Y	8/8	100
(Ratcliffe & Smith, 2014) [72]	Y	Y	Y	Y	U	U	Y	Y	6/8	75
(Zimmerman et al., 2020) [49]	Y	Y	Y	Y	Y	U	Y	Y	7/8	87.5
(Choi & Kim, 2018) [24]	Y	Y	Y	Y	Y	Y	Y	Y	8/8	100
(van Gulik et al., 2021) [29]	Y	Y	Y	Y	Y	Y	Y	Y	8/8	100
(Hernandez-Garcia & Cardoso, 2013) [45]	U	U	Y	Y	N	N	Y	Y	4/8	50
(Bargellini et al., 2014) [21]	Y	Y	Y	Y	Y	Y	Y	Y	8/8	100
(Skodova et al., 2014) [44]	U	U	Y	Y	U	U	Y	Y	4/8	50
(Singh & Barnard, 2023) [68]	Y	Y	Y	Y	Y	Y	Y	Y	8/8	100
(Maras et al., 2024) [57]	Y	Y	Y	Y	Y	Y	Y	Y	8/8	100
(Jeong & Kim, 2016) [15]	Y	Y	Y	Y	Y	Y	Y	Y	8/8	100
(Darawad & Al-Hussami, 2013) [60]	Y	Y	Y	Y	Y	Y	Y	Y	8/8	100
(Ojulong et al., 2013) [36]	Y	U	Y	Y	Y	Y	Y	Y	7/8	87.5
(Brosio et al., 2017) [47]	Y	Y	Y	Y	Y	Y	Y	Y	8/8	100
(Khubrani et al., 2018) [35]	Y	Y	Y	Y	Y	Y	Y	Y	8/8	100
(Majidipour et al., 2019) [42]	Y	Y	Y	Y	Y	Y	Y	Y	8/8	100
(Khan et al., 2021) [16]	Y	Y	Y	Y	U	U	Y	Y	6/8	75
(Thakker & Jadhav, 2015) [37]	Y	Y	U	Y	U	U	Y	Y	5/8	62.5
(Saurabh et al., 2022) [66]	U	Y	Y	U	U	U	Y	Y	4/8	50
(Nair et al., 2014) [38]	U	U	Y	Y	Y	U	Y	U	4/8	50
(Sharma & Bachani, 2023) [7]	Y	Y	Y	Y	U	U	Y	Y	6/8	75
(Khan et al., 2021) [16]	Y	Y	Y	Y	Y	U	Y	Y	7/8	87.5
(Oh, 2021) [43]	Y	Y	Y	Y	Y	Y	Y	Y	8/8	100
(Hung et al., 2017) [61]	Y	Y	Y	Y	Y	Y	Y	Y	8/8	100
(de Lourdes Lopes et al., 2023) [63]	Y	Y	Y	Y	Y	Y	Y	Y	8/8	100
(AL-Rawajfah & Tubaishat, 2015) [39]	Y	Y	Y	Y	Y	Y	Y	Y	8/8	100
(Cheung et al., 2015) [54]	Y	Y	Y	Y	Y	Y	Y	Y	8/8	100
(Cruz & Bashtawi, 2016) [25]	Y	Y	Y	Y	Y	Y	Y	Y	8/8	100
(Kingston et al., 2017) [26]	Y	Y	Y	Y	Y	Y	Y	Y	8/8	100
(Sundal et al., 2017) [69]	Y	Y	U	U	Y	U	U	Y	4/8	50
(Baba et al., 2023) [46]	U	U	Y	Y	U	U	Y	Y	4/8	50
(Erwin et al., 2026) [51]	Y	Y	Y	Y	U	U	Y	Y	6/8	75
(Kingston et al., 2018) [27]	Y	Y	Y	Y	Y	Y	Y	Y	8/8	100
(Syed & Al-Rawi, 2024) [70]	Y	Y	Y	Y	U	U	Y	Y	6/8	75
(Blomgren et al., 2024) [64]	Y	Y	Y	Y	Y	Y	Y	Y	8/8	100
(Linnik et al., 2024) [28]	Y	Y	Y	Y	Y	U	Y	Y	7/8	87.5
(Coşkun Palaz & Demir Erbaş, 2025) [59]	Y	Y	Y	Y	U	U	Y	Y	6/8	75
(Yıldız et al., 2024) [48]	Y	Y	Y	Y	U	U	Y	Y	6/8	75
(Ntaote et al., 2024) [56]	Y	Y	Y	Y	Y	Y	Y	Y	8/8	100

Y: yes; N: no or not applicable; U: unclear.
